# Evaluation of Outbreak Preparedness to Prevent the Reintroduction of Malaria in Ramanagara District: A Descriptive Epidemiological Study

**DOI:** 10.7759/cureus.63162

**Published:** 2024-06-25

**Authors:** Chaithra S, Ranganath TS, Saraswathi S, Ravikumar K, Dhaheera Dheeshan, Shambhavi A Vaidya, Lakshmikanth N

**Affiliations:** 1 Department of Community Medicine, Bangalore Medical College and Research Institute, Bengaluru, IND; 2 Public Health, Independent Consultant, Bengaluru, IND

**Keywords:** malaria elimination, outbreak preparedness, retrospective evaluation, reintroduction, indigenous, vector control

## Abstract

Background

Malaria is a vector-borne disease transmitted by female anopheline mosquitoes. It is also a multidimensional disease influenced by social factors such as poor environmental conditions and awareness gaps. India has witnessed a substantial reduction in malaria cases and has declared three regions as malaria-free, with Karnataka being one of the states. However, Karnataka witnesses significant population movement and migration, which influences the spread of malaria. Ramanagara, a district in Karnataka, reported zero indigenous cases over the past three years. Hence, we selected this district to evaluate outbreak preparedness to prevent the reintroduction of malaria. This choice underscores the district’s significance as a valuable model for preventing the reintroduction of malaria.

Methodology

Baseline survey data on malaria cases and vector survey data were evaluated for the period spanning 2018 to 2022. The data were gathered from both the regional office and the Ramanagara district health office. In addition to the documenting system, because there was no regular submission of Form-P and Form-L of the Integrated Disease Surveillance Project and Integrated Health Information Platform from the private sector, to complete the missing data, a cross-sectional study was conducted among private sector practitioners and pharmacies in Ramanagara from April 2023 to June 2023. Data was collected via interviews using a malaria surveillance assessment toolkit sourced from the National Centre for Vector Borne Diseases Control and World Health Organization protocols to assess six core areas of malaria elimination. Data collected via interviews were compiled in MS Excel and analyzed using SPSS Statistics for Windows, version 26.0 (IBM Corp., Armonk, NY, USA).

Results

Malaria control measures in the selected district achieved >80% coverage with notable improvements in the National Health Mission-National Vector Borne Disease Control Programme fund utilization, logistics availability, and physical performance over the past five years. The Annual Parasite Index was <1 at 0.0061 in 2018 and 0.0017 in 2022. The annual blood examination rate was consistently >10 from 22.05 in 2018 to 24.36 in 2022. The primary vectors identified were *Anopheles culicifacies* and *Anopheles stephensi*. In 2018, there were six cases of *Plasmodium vivax* and one case of *Plasmodium falciparum* reported as imported cases. In 2021 and 2022, two cases of *P. vivax* were reported. Notably, there were no instances of mixed infections or indigenous cases documented from 2018 to 2022

Conclusions

Although the level of outbreak preparedness in the region is satisfactory, the effectiveness of vector control measures appears to be lacking. Increased government funding is needed along with comprehensive training and workshops for healthcare workers. Adequate financial resources and enhanced skills among healthcare workers are crucial to reinforce the existing efforts to control vectors and prevent potential outbreaks effectively.

## Introduction

Globally, in 2020, malaria cases reached 241 million, with India accounting for 1.7% of cases and 1.2% of related deaths [1]. Remarkably, the implementation of India’s National Strategic Plan for Malaria Elimination has led to a significant reduction in disease burden with a decline in malaria-related morbidity and mortality [2], contributing to the country’s progress toward Sustainable Development Goal 3.3 [3]. Despite significant reductions, the risk of re-emergence persists due to complexities associated with unplanned urbanization [4], the presence of endemic pockets in remote areas, increased migration, seasonal variations, and climate change [5], which often lead to the disruption of anti-malarial measures in previously controlled regions of endemicity. India has been in the phase of malaria elimination [6], with several regions reporting zero indigenous cases. The most difficult issue that the country currently confronts in its quest to eliminate malaria is increased population density in both urban and semi-urban settings [7,8]. To tackle these problems, close entomological monitoring [9], effective surveillance, implementation of stringent preventive measures, and community engagement are vital. The primary objective of public health is the timely detection and prevention of impending epidemics. Keeping this rationale in mind, this study seeks to provide a crucial insight into the current status of outbreak preparedness and vector control measures implemented at the grassroots level in the Ramanagara district of Karnataka.

## Materials and methods

An evaluation of baseline survey data for malaria cases along with vector survey data was conducted from 2018 to 2022. The data were gathered from both the regional office and the Ramanagara district health office. Data on confirmed malaria cases and vector surveys were collected by investigators during survey and field visits in the low-coverage areas of Ramanagara district.

Paper-based annual reports, district data, and epidemiological data were gathered. The primary health centers reported fever incidences and malarial cases on the M1 and M2 forms of the National Vector Borne Disease Control Programme and Form P and Form L of the Integrated Disease Surveillance Project (IDSP). However, the coverage from the IDSP was poor. Because there was insufficient data from the private sector to complete the missing data and conduct cross-verification, after obtaining informed consent, a cross-sectional study was conducted from April to June 2023 among the six private sector practitioners and pharmacies of Ramanagara district.

All individuals who underwent blood smear examination (1,194,346) between 2018 and 2022, including migrants, and all malaria cases diagnosed by a rapid diagnostic test or microscopically confirmed cases were included in the study and were selected using universal sampling to ensure comprehensive representation. The malaria surveillance assessment toolkit sourced from the World Health Organization (WHO) [10] was used to assess six core areas in alignment with the national malaria elimination assessment committee protocol, as specified by the National Centre for Vector Borne Disease Control (NCVBDC) and the WHO for subnational verification (Table 1).

**Table 1:** Areas of assessment and their components.

Areas of assessment	Components
Program capacity to deliver services	Surveillance (epidemiological and entomological), diagnosis, and case management
Vector control	Insecticide resistance monitoring, use of long-lasting insecticidal nets (LLINs), and ensuring the quality of insecticide residual spraying (IRS)
Periodicity and quality assurance of reporting as per National Centre for Vector Borne Diseases Control (NCVBDC) formats	Monthly
Adequacy of human resources and their capacity	Training and capacity building
Availability and use of funding for the activities for which it was released	Funds for mobilization, training and vector control, and prevention measures
System in place for sustaining surveillance and vector prevalence	Mapping of water bodies, exploring seasonal trends and areas of outbreak occurrence

Statistical analysis

Data were collected and entered into MS Excel and analyzed using SPSS Statistics for Windows, version 26.0 (IBM Corp., Armonk, NY, USA). The analyzed data are presented using appropriate tables and graphs.

Ethical considerations

Approval and clearance were obtained from the Institutional Ethics Committee of Bangalore Medical College and Research Institute (approval number: BMCRI/EC/20/23-24), and permission was received from the concerned authorities.

## Results

According to the malaria elimination criteria set by the WHO for program phasing, the “elimination phase” (Category 1) is designated when the Annual Parasite Index (API) is less than 1 case per 1,000 population at risk. A district with an API <1 and an annual blood examination rate (ABER) >10 is considered to be in the elimination phase.

Epidemiological surveillance

Table 2 illustrates the progressive decline in API indices. The API decreased from 0.0061 in 2018 to 0.0017 in 2022, and the ABER increased from 22.05 in 2018 to 24.36 in 2022. There was a decline in the ABER in 2020, but the overall ABER remained above 10 over the past five years. Among the screened population, the slides examined via blood smear examination showed *Plasmodium falciparum* and *Plasmodium vivax*. The total malaria cases decreased from seven (2018) to two (2022) with no mixed infection. An epidemiological investigation conducted by the district vector-borne disease control office showed that all these positive cases were imported cases. There were no indigenous cases.

**Table 2:** Program capacity: malaria metric indices. API = Annual Parasite Index; ABER = annual blood examination rate; SPR = slide positivity rate; PV = *Plasmodium vivax*; PF = *Plasmodium falciparum*

Year	Population	Blood Smear Examined	API	ABER	SPR	PV	PF	Total mix cases	Indigenous	Total positives: all imported (SPR)
2018	1,142,338	251,928	0.0061	22.05	0.0028	6	1	0	0	7
2019	1,130,979	264,881	0.0044	23.4	0.0018	4	1	0	0	5
2020	1,146,549	171,593	0.0026	17.0	0.0017	2	1	0	0	3
2021	1,159,118	226,043	0.0017	19.4	0.0008	2	0	0	0	2
2022	1,148,963	279,901	0.0017	24.36	0.0007	2	0	0	0	2

Vector control logistics

Available vector controls were available in desired quantities and included long-lasting insecticidal nets (LLINs); insecticides such as dichlorodiphenyltrichloroethane (DDT) 50%, soluble powder (SP) 10% and 5%, and malathion 95%; and SP adulticides such as temephos 50%, pyrethrum extract 2%, deltamethrin 2.5% (liquid), and cyphenothrin 5%. Spray pumps were also present.

Available anti-malarial drugs included chloroquine phosphate 250 mg (150 mg base), primaquine phosphate 2.5 mg, primaquine phosphate 7.5 mg, quinine sulfate, Combi Pack (CQ + PQ), artemisinin-based combination therapy (ACT), and Combi Blister Pack (pediatrics) for 0-1 year, 1-4 years, 5-8 years, and 9-14 years. Artemether, artesunate, quinine injection, and RD kits for malaria (bivalent) were also available.

Periodicity and quality assurance of reporting

NCVBDC reports showed decreased reporting from 136 to 50 between January 2022 and March 2023. Overall, the coverage of malaria diagnosis and treatment was above 80% according to monthly reporting data in the NVBDC format. The blood smears from various primary health centers (PHCs) of Ramanagara district were initially examined by PHC technicians, and the declared negative samples were re-examined by nodal laboratory technicians. Subsequently, these negative blood smears were brought to the district level for further examination. Upon scrutiny at the district level, no discrepancies were found.

Table 3 shows imported and migratory cases of malaria. In 2019, five positive cases were detected in Ramanagara district. Migratory screening reduced the reports from 4,283 in 2018 to 1,070 in 2022, of which two were positive on rapid diagnostic tests.

**Table 3:** Migratory screening in Ramanagara district.

Year	Total migrant population	Migrants screened for Malaria, n (%)
2018	4,399	4,283 (97.36)
2019	1,255	903 (71.95)
2020	658	428 (65.04)
2021	1,404	1,404 (100)
2022	1,070	1,070 (100)

Table 4 illustrates that Ramanagara district has 69 PHCs, of which 56 had adequate human resources, monocular and binocular microscopes, blood lancets, and glass slides. In the remaining 13 PHCs, microscopists were not available. The quality of the slides prepared by auxiliary nurse midwives and multipurpose workers must be improved to achieve quality assurance. Adequate training and reorientation for all healthcare workers is necessary.

**Table 4:** Adequacy and capacity of human resources.

Name of Taluk	Number of primary health centers	Number of microscopists	
		In position, n (%)	Vacant, n (%)
Channapattana	16	13 (81.25)	03 (18.75)
Kanakapura	21	16 (76.19)	05 (23.81)
Magadi	15	11 (73.34)	04 (26.66)
Ramanagara	17	16 (94.12)	01 (5.88)
Total	69	56 (81.15)	13 (18.85)

Table 5 shows the approved funds for the malaria program and their utilization across its four components. The ASHA Incentives component initially experienced a decline in utilization. However, it later recovered and achieved full utilization in 2021-2022. The Monitoring, Evaluation, and Supervision component initially had an increase in utilization but dropped to 73.93% in 2021-2022. The Mobility for Epidemic Preparedness component demonstrated fluctuations in utilization. Meanwhile, the Governance Cost of NAMMIS component consistently exhibited a steady increase over the period.

**Table 5:** Availability and utilization of funds for activities: financial performance for malaria elimination. Approved and utilized funds (in INR) *: Based on the web-based National Anti-Malaria Management Information System (NAMMIS). API = Annual Parasite Index; ABER = annual blood examination rate; SPR = slide positivity rate

Components	2018–2019			2019–2020		2020–2021		2021–2022	
	Approved		Utilized	Approved	Utilized	Approved	Utilized	Approved	Utilized
ASHA Incentives	125,000		125,000 (100%)	125,000	105,480 (84.38%)	50,000	43,260 (86.52%)	25,000	25,000 (100%)
Monitoring, Evaluation, and Supervision	1,64,805		122,000 (74.03%)	36,000	32,600 (95.56%)	75,000	74,815 (99.75%)	100,000	73,927 (73.93%)
Mobility for Epidemic preparedness	-		-	70,000	69,709 (99.58%)	50,000	33,278 (66.56%)	50,000	48,651 (97.30%)
Governance Cost of NAMMIS*	-		-	-	-	24,000	12,000 (50%)	24,000	22,000 (91.67%)
Paired samples t-test variables					Statistic value		df		P-value
API		ASHA Incentives			-5.19		3.00		0.014
		Monitoring, Evaluation, and Supervision			-5.18		3.00		0.014
		Mobility for Epidemic preparedness			-5.18		3.00		0.014
		Governance Cost of NAMMIS			-5.19		3.00		0.014
ABER		ASHA Incentives			13.35		3.00		<0.001
		Monitoring, Evaluation, and Supervision			12.80		3.00		<0.001
		Mobility for Epidemic preparedness			14.16		3.00		0.001
		Governance Cost of NAMMIS			13.35		3.00		<0.001
SPR		ASHA Incentives			-5.19		3.00		0.014
		Monitoring, Evaluation, and Supervision			-5.19		3.00		0.014
		Mobility for Epidemic preparedness			-5.19		3.00		0.014
		Governance Cost of NAMMIS			-5.19		3.00		0.014
Blood smear examined		ASHA Incentives			11.07		3.00		0.002
		Monitoring, Evaluation, and Supervision			11.07		3.00		0.002
		Mobility for Epidemic preparedness			11.07		3.00		0.002
		Governance Cost of NAMMIS			11.07		3.00		0.002

Regarding entomological surveillance, we also reviewed the entomological data of the previous year. Earlier studies were taken from the entomological logistics and analyzed. The incidence of malaria cases was recorded in the months from May to July 2022. The primary vectors found in the Ramanagara district were *Anopheles culicifacies* and *Anopheles stephensi*, and the secondary vectors were *Anopheles subpictus*, *Anopheles annularis*, and *Anopheles varuna*. In Ramanagara district, no indigenous malaria cases have been reported in the past three years. Major outbreaks occurred in 2010 and 2011. Around 232 malaria cases were reported in 32 quarry areas belonging to Ramanagara district. This outbreak was mainly due to labor migration from endemic districts to the Bidadi quarry area. As a result, an entomological study was conducted in 126 villages from 2015 to 2022. In 51 villages or areas, dawn and dusk collections were done in cattle sheds, and in 75 villages or areas, daytime collections were done in human dwellings. *A. culicifacies* mosquitoes were found in low density in Kokkedodddi, Kodipura, Vaderahalli, Chandurayanahalli, Allimaranahalli, Yeligehalli Colony, Bairapattana, and in two villages, namely, Vajarahalli Colony and Yarabnagar. *A. stephensi* mosquitoes were found in a lower density during the study as well as during routine larval surveys. Areas around Bidadi quarry were also visited routinely to look for breeding sources. No breeding places and water stagnation were seen in the quarry areas. Bidadi Industrial Area and Harohalli Industrial Area experienced seasonal variations and outbreaks due to percolations of water and interrupted vector control measures.

**Figure 1:**
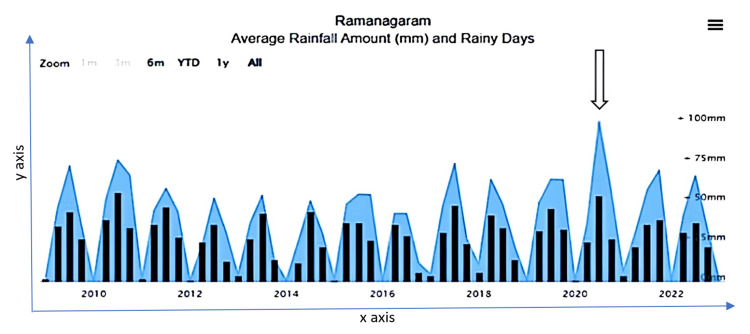
Annual average rainfall amounts and rainy days in Ramanagara district. x-axis: monthly and yearly rainfall distributions. y-axis: average rainfall amounts (mm). Source: Annual reports on seasonal trends, District Health Office, Ramanagara District (2022).

Figure 1 illustrates the seasonal trends in rainfall changes in the eco-epidemiological system. The dry season occurs from January to February, followed by hot weather from March to May. The southwest monsoon season is from June to September and the northeast monsoon period is from October to December. The amount of rainfall is relatively uniform throughout the district. The period from December to March represents the very low rainfall months. Approximately 100 mm of average rainfall was noticed in 2020, with rainfall occurring on nearly 49 rainy days a year. Among the water bodies in Ramanagara district are one reservoir, 22 fishery department tanks, and 56 Gram Panchayat tanks.

**Figure 2:**
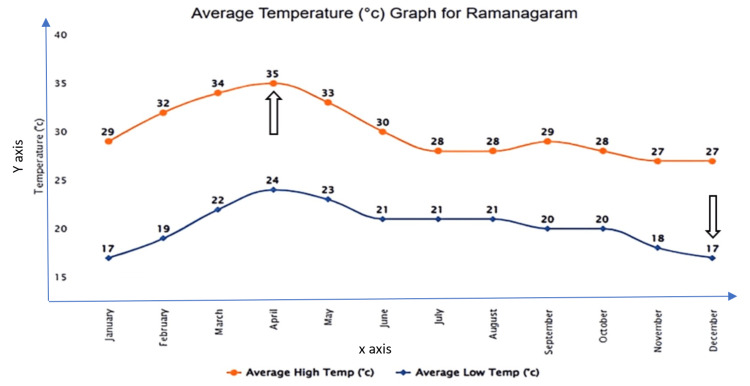
Average temperatures in the Ramanagara district. x-axis: months of the year; y-axis: average temperature in °C. Source: Annual reports on seasonal trends, District Health Office, Ramanagara District (2022).

Figure 2 shows that the temperature starts rising in January and peaks in April, with a maximum temperature of 35°C. Thereafter, it declines during the monsoon period. December is the coldest month with the temperature dipping down to 17°C. The humidity is lowest during the dry season and highest during the monsoon period. The wind direction is predominantly southwesterly during the summer monsoon and northeasterly during the winter monsoon.

We formally requested information on the seasonal trends of Ramanagara district from the relevant authorities. They officially provided these documents to us via email, implying permission.

Both government and private healthcare facilities must report all positive malaria cases and should screen 15% of all fever cases for malaria parasites, as mandated. However, there was a disparity in slide preparations in the private sector, possibly because of a reliance on rapid diagnostic testing. Even though there were more fever cases in private setups in Ramanagara district, the rate of blood smear examinations was comparatively low. In government facilities, the number of slides examined was unsatisfactory and requires quality improvement.

For the Malaria Elimination Survey in Ramanagara, two private practitioners reported that there were no positive cases based on blood smears in the past three years. In Magadi, one of the talukas of the Ramanagara district, three private practitioners reported two cases, one a positive rapid diagnostic test and the other a positive blood smear (*P. vivax*). Both were identified as migrant cases. Laboratory technicians in private settings lack adequate training for malaria blood smear examination, and cases are confirmed by pathologists at higher centers. It is crucial to train these technicians to augment the annual blood examination rate at private healthcare centers.

## Discussion

Krishna et al. (2017) conducted a retrospective case analysis in Tumkur that revealed a steady decline in malaria cases from 27,553 to 68 with a decreasing trend in API. Similarly, they also showed that the average ABER was 23.23% with no reported fatalities over the last 15 years (2001-2015) and no outbreaks in the past three years. The study findings indicated a seasonal trend, with an increased incidence of malaria cases in the rainy season from June to October [11]. In this study, the API reduced from 0.0061 in 2018 to 0.017 in 2022, and the ABER increased from 22.05 in 2018 to 24.36 in 2022 with zero indigenous cases and no reported fatalities in the past five years (2018-2022). The incidence of malaria cases was reported from May to July. It has been noted that the passive screening of malaria cases decreased during the COVID-19 pandemic in 2020. In both Ramanagara and Tumkur, the API and ABER showed a similar pattern.

A study by Kirinyet et al. in Eldoret West district revealed variations in the reporting rates from health sectors in the Integrated Disease Surveillance and Response system. Health facilities such as government-owned facilities had higher reporting rates compared to private, mission, or NGO-based facilities (45.8%). Although 51% of health facilities had a reporting rate of at least 90%, some facilities, especially private and mission hospitals, reported rates below 50% [12]. Similarly, in Ramanagara district, even though both private and government healthcare sectors were given the goal of screening all fever cases for malaria and 15% of all fever cases from the new outpatient department for malaria as per the state authorities, performance from the private sector was comparatively lower. The gold standard for malaria diagnosis is the microscopic examination of a blood smear. The government health centers screen almost all fever cases by blood smear examination, whereas in the private sector, healthcare providers do not perform smears regularly and instead use rapid diagnostic testing more frequently for the diagnosis of malaria.

Dayanand et al.’s (2014) cross-sectional surveillance in Mangaluru highlighted higher infection rates among male adults and immigrant laborers, correlating with literacy levels, knowledge, and dwelling conditions. The study emphasized the significance of improving preventive measures and hygiene awareness, especially in hotspot areas with high vector density [13]. This study revealed that over the years, the migratory screening of the indigenous population migrants has resulted in reductions in malaria cases from 4,283 in 2018 to 1,070 in 2022 in the Ramanagara district. Imported cases of malaria were identified in Ramanagara district, specifically among migrant laborers working in stone crushers, out of which two were positive on rapid diagnostic tests, with no positive cases in the indigenous population.

The editorial by Narain and Nath (August 2018) emphasized India’s ambitious target for malaria elimination. It stressed the importance of addressing the shortage of trained healthcare workers and fostering comprehensive community engagement and called for a paradigm shift in surveillance, program management, and resource allocation to meet the elimination goal [14]. According to this study, the Ramanagara district lacks laboratory technicians, multipurpose workers, and auxiliary nurse midwives. The quality of the slides for malarial parasite examination must be improved to achieve the quality assurance for which the ongoing training of healthcare professionals is crucial. In both studies, the potential positive effects of improved training on the accuracy and reliability of malaria diagnosis were emphasized.

A study by Smith Gueye et al. outlined vector control interventions, including integrated vector management, but their impact remains uncertain. Although entomological surveillance was prevalent, the response lacked specificity. Solutions include limiting stratification and increasing targeted interventions, outbreak forecasting, and evidence-based real-time vector control response strategies. Malaria resurgence resulted from halted indoor residual spraying (IRS) coverage, often due to funding and operational constraints and poor implementation. Although bed net usage was widespread, measurements of coverage and effectiveness were inadequate. Larval control is crucial for some countries, but details are lacking regarding coverage and impact. Inconsistent coverage indicators and tool selection suggested that decisions were influenced more by funding, operational challenges, and costs than by evidence-based effectiveness [15]. In this study, the funds dedicated to the malaria program under the national health mission for certain components have increased from INR 119,760 (2017-2018) to INR 194,408 (2021-2022). However, adequate training for the utilization of vector logistics was lacking.

A study done by Gayan Dharmasiri et al. (2017) concluded that the initial documentation of *A. stephensi* in Mannar Island, Northern Sri Lanka, posed a potential threat to malaria prevention. *A. stephensi* is abundant in larval habitats (36.65%) and coexists with other species. Despite Sri Lanka’s WHO-certified malaria-free status, the presence of* A. stephensi* poses a serious challenge to the Ministry of Health’s efforts, particularly in urban and vulnerable areas, potentially facilitating malaria reintroduction. This necessitates sustained vigilance, financial support, and comprehensive preventive measures to avert potential resurgence. Their findings showed the importance of understanding vector biology and genetic variations to prevent future re-establishment [16]. In Ramanagara district, entomological surveillance has revealed the presence of vectors such as *A. culicifacies* and *A. stephensi*. Their presence continues to pose a challenge to the certification of malaria elimination in the area and remains an obstacle to preventing the reintroduction of malaria into that region.

Dhiman et al. conducted a study (2010-2013) in Uttarakhand that showed that increased temperatures extended transmission seasons, elevating *A. culicifacies* and *A. fluviatilis* density, underscoring the need for tailored preparedness and heightened surveillance against climate-induced shifts [17]. The entomological data showed the different levels of vector density in Ramanagara during the monsoon season were caused by the percolation of water, availability of temporary settlements, migratory population, and open drainage system.

Study limitations include the inability to collect data from all practitioners and pharmacies of Ramanagara district because of resource constraints. Private sector surveillance was incomplete because of limited time and resources, and the number of PHC visits was restricted because of time limitations. Furthermore, research articles with similar objectives and research questions are few, so effective comparisons could not be established.

## Conclusions

The evaluation of outbreak preparedness showed that further strengthening of the system is necessary to prevent future epidemics. The healthcare workers in place are trained to identify any outbreak. A rapid response team at the district level was established to assess the outbreak preparedness to prevent the reintroduction of malaria and assess the vector control measures. The ABER is consistently monitored district-wide, both at the institutional and PHC levels. The quantum of surveillance is good. The migratory population is screened regularly. An appropriate reporting system is in place through the IDSP and Integrated Health Information Platform. The possibility of reintroduction cannot be ruled out because of the migrant population, imported cases, and the presence of the vector. Therefore, better quality implementation and efficient training and reorientation for healthcare professionals are needed. The study findings indicate a significant decline in malaria cases in Ramanagara district over the past five years, with no indigenous cases reported. Despite adequate availability of vector control logistics and anti-malarial drugs, challenges remain in terms of slide preparation quality and blood smear examination rates, particularly in private healthcare facilities. Ensuring comprehensive training for laboratory technicians and promoting standardized diagnostic procedures across all healthcare settings are essential for sustaining malaria elimination efforts in the district.

To prevent the re-establishment of malaria, sustained surveillance for vector species and effective control of imported cases through rapid detection and early diagnosis are required. A malaria vigilance system needs to be carefully planned and well managed to ensure early recognition and prompt control of introduced cases that might lead to an epidemic. Every case of transfusion malaria from known or suspected blood donors should be routinely reported and followed up. To strengthen vector control, insecticide resistance must be monitored and the coverage of LLINs and IRS must be intensified in peripheral and low-coverage areas. A thorough survey should be done to map the quarries as well as the migrant population and to identify and monitor all sources of water for vector breeding places year-round. Information education communication should be intensified to engage community participation in preventing vector-borne diseases and increase the knowledge of malaria and its control in the community.
